# Proteomics-Based Serum Alterations of the Human Protein Expression after Out-of-Hospital Cardiac Arrest: Pilot Study for Prognostication of Survivors vs. Non-Survivors at Day 1 after Return of Spontaneous Circulation (ROSC)

**DOI:** 10.3390/jcm11040996

**Published:** 2022-02-14

**Authors:** Jochen Hinkelbein, Lydia Kolaparambil Varghese Johnson, Nikolai Kiselev, Jan Schmitz, Martin Hellmich, Hendrik Drinhaus, Theresa Lichtenstein, Christian Storm, Christoph Adler

**Affiliations:** 1 Department of Anesthesiology and Intensive Care Medicine, Faculty of Medicine, University Hospital Cologne, 50937 Cologne, Germany; jan.schmitz1@uk-koeln.de (J.S.); hendrik.dinhaus@uk-koeln.de (H.D.); 2 Faculty of Medicine and Surgery, Università degli Studi di Perugia, 05100 Terni, Italy; lydia.kolaparambil@gmail.com; 3 Clinic for Anesthesiology, Intensive Care Medicine, Preclinical Emergency Medicine and Pain Management, Sankt Katharinen Hospital Frechen, 50226 Frechen, Germany; n.kiselev@gmx.net; 4 Institute of Medical Statistics and Computational Biology (IMSB), Faculty of Medicine, University Hospital Cologne, University of Cologne, 50937 Cologne, Germany; martin.hellmich@uk-koeln.de; 5 Department of Psychiatry and Psychotherapy, Faculty of Medicine, University Hospital Cologne, University of Cologne, 50937 Cologne, Germany; theresa.lichtenstein@uk-koeln.de; 6 Medical Department, Division of Nephrology and Internal Intensive Care Medicine, Charité—Universitaetsmedizin Berlin, 10117 Berlin, Germany; christian.storm@charite.de; 7 Heart Centre, University Hospital Cologne, 50937 Cologne, Germany; christoph.adler@uk-koeln.de; 8 Fire Department City of Cologne, Institute for Security Science and Rescue Technology, 50737 Cologne, Germany

**Keywords:** proteomics, CPR, protein expression, targeted temperature management, ROSC

## Abstract

Background: Targeted temperature management (TTM) is considered standard therapy for patients after out-of-hospital cardiac arrest (OHCA), cardiopulmonary resuscitation (CPR), and return of spontaneous circulation (ROSC). To date, valid protein markers do not exist to prognosticate survivors and non-survivors before the end of TTM. The aim of this study is to identify specific protein patterns/arrays, which are useful for prediction in the very early phase after ROSC. Material and Methods: A total of 20 adult patients with ROSC (19 male, 1 female; 69.9 ± 9.5 years) were included and dichotomized in two groups (survivors and non-survivors at day 30). Serum samples were drawn at day 1 after ROSC (during TTM). Three panels (organ failure, metabolic, neurology, inflammation; OLINK, Uppsala, Sweden) were utilised. A total of four proteins were found to be differentially regulated (>2- or <−0.5-fold decrease; *t*-test). Bioinformatic platforms were utilised to analyse pathways and identify signalling cascades and to screen for potential biomarkers. Results: A total of 276 proteins were analysed and revealed only 11 statistically significant protein alterations (Siglec-9, LAYN, SKR3, JAM-B, N2DL-2, TNF-B, BAMBI, NUCB2, STX8, PTK7, and PVLAB). Following the Bonferroni correction, no proteins were found to be regulated as statistically significant. Concerning the protein fold change for clinical significance, four proteins (IL-1 alpha, N-CDase, IL5, CRH) were found to be regulated in a clinically relevant context. Conclusions: Early analysis at 1 day after ROSC was not sufficiently possible during TTM to prognosticate survival or non-survival after OHCA. Future studies should evaluate protein expression later in the course after ROSC to identify promising protein candidates.

## 1. Introduction

Sudden cardiac arrest is a sword of Damocles causing around 20% of all deaths [1] in the world. Additionally, each year, 375,000 people in Europe [2] require immediate cardiopulmonary resuscitation (CPR) after out-of-hospital cardiac arrest (OHCA). Targeted temperature management (TTM) is considered as a grade IB recommendation [3] since 2010, as it improves mortality and the neurological outcomes significantly after the return of spontaneous circulation (ROSC). The case-fatality rate for patients after ROSC is still very high, arriving at a rate of around 71.5% after 1 year [4].

In this context, prognostication and predicting the outcome is of cardinal importance since brain injury is the determinant of morbidity and mortality in these patients [5]. According to the 2015 ERC guidelines [6], the earliest time to predict a poor neurological outcome using clinical examination in comatose patients is 72 h after cardiac arrest or 72 h after the restoration of normothermia in patients treated with TTM.

The majority of mortality after ROSC is due to hypoxic–ischemic brain injury (HIBI) [7]. In addition, a good prognostication is essential to minimise a falsely pessimistic prediction in comatose patients [8]. To date, multiple prognostic tests were evaluated for neurological prognostication [5], such as cranial computer tomography (CCT), detailed clinical neurological assessment, electroencephalography (EEG), and measurement of somatosensory-evocable potentials (SEPs). Nonetheless, these are not always accurate predictors for the neurological outcome and specifically for survival for several reasons, such as the sedation for induction. In addition, the maintenance of TTM decreases the validity of prognostication [7]. Enolase-2 (NSE) or S-100B serum markers are other possible methods. However, these markers alone do not provide a valid prognostication of the clinical outcome since they are influenced by multiple factors [9]. Ulterior limitations are based on the fact that prognostication cannot be made prior to the return of normothermia [6]. Additionally, to date, other reliable single-protein markers do not exist to prognosticate survivors and non-survivors prior to the end of TTM. However, the current guidelines recommend a multimodal strategy for prognostication.

In this prospective cohort study, serum proteins of survivors and non-survivors of OHCA are analysed by both proteomic and bioinformatic methods to identify proteins of interest, which could allow for prognostication if used in an array or as a set of proteins. This study aims to investigate the proteome in the context of TTM after cardiac arrest and to identify specific protein patterns that are employable in prognostication, which could be useful as a complete set for estimating survival.

## 2. Material and Methods

The main goal of this study was to identify protein markers that are useful for clinical outcome prediction in the early phase of treatment (i.e., 24 h after ROSC and during TTM) in patients after CPR and ROSC.

### 2.1. Study Design

This prospective observational study included 20 patients with cardiac causes, resuscitated from a non-traumatic, non-hypoxic-related, out-of-hospital cardiac arrest (OHCA), and treated with TTM, according to the standard protocol of the hospital for at least 24 h.

### 2.2. Patients

Adult patients, admitted for an OHCA after CPR, ROSC, and TTM were included in the study. Patients presenting hypoxia-related, traumatic or other causes were excluded from the study.

### 2.3. Sample Collection

**Figure 1:**
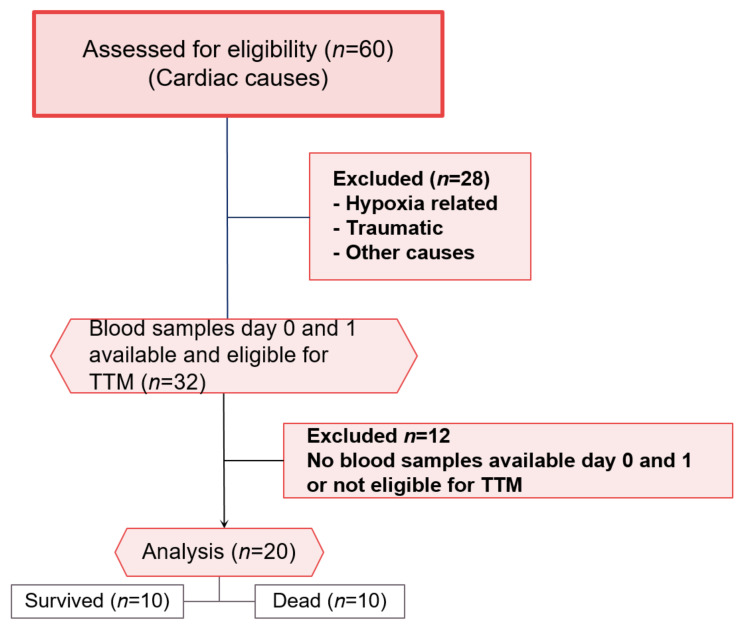
CONSORT flow chart for patients included and excluded in the study. Surviving and diseased patients (*n* = 10) were analysed.

In all patients, blood was drawn on day 0 (i.e., the day of CPR in the emergency department or ICU after arrival) and day 1 (i.e., after 24 h and during TTM). If no blood sample was available for one of both days, patients were excluded from the analysis (Figure 1). Patients that are not eligible for TTM were also excluded from the study. Survivors and non-survivors were dichotomized on the 30th day after CPR to allocate patients to the two compared groups: Survivors vs. non-survivors.

For all (*n* = 20) patients, demographic and clinical variables were collected from the electronic files of the patients via the ORBIS software (AGFA HealthCare, Bonn, Germany). Blood samples were collected daily at the same time from an arterial line into serum tubes. After the acquisition, the serum was centrifuged at 5000× *g* for 5 min and stored at −80 °C until proteomic analysis at the end of the study.

### 2.4. TTM Therapy

In clinical routine, the ERC guideline recommendations [10] for the treatment of cardiac arrest and post-cardiac arrest care management were utilised. Briefly, patients were treated with TTM for 24 h, according to our standard operating procedure (SOP). The emergency medical service initiated peripheral cooling with ice packs to the femoral and/or neck area in patients with OHCA. The controlled cooling to a target temperature of 32–34 °C was continued in the intensive care unit (ICU) using an endovascular cooling device (Thermogard XP^®^ catheter, Zoll Medical Corp., Chelmsford, MA, USA) and maintained for 24 h. TTM was terminated by rewarming through the same endovascular device at a controlled rate of 0.3 °C/h until the physiologic body temperature of 36.5 °C was reached. This temperature was maintained for further 48 h. The basic metabolic panel, magnesium, phosphorus, ionized calcium, CBC with differential, and PT/PTT were monitored every 6 h during the clinical routine.

### 2.5. Proteomic Analysis

The concept of proteomic and biostatistical analysis of proteins is defined as the separation, identification, and quantification of the entire protein of a cell, organism or tissue under specific conditions. Cardiac arrest leads to a critical whole body ischemia and in the case of ROSC, additional damage occurs during and after reperfusion. The so-called Post-Cardiac Arrest Syndrome is a combination of pathophysiological processes, which is associated with post-cardiac arrest brain injury, post-cardiac arrest myocardial dysfunction, and systemic ischemia/reperfusion response. To improve the complex interaction between the different organ systems, we decided to choose the following panels: Inflammation panel, organ damage panel, and neurology panel.

In summary, as a first step, statistically significantly regulated proteins were identified by OLINK and analysed by bioinformatic network analyses (GeneMania^®^, Toronto, ON, Canada; http://www.genemania.org, accessed on 14 December 2021). Thereafter, these statistically significant proteins were grouped using a hierarchical cluster analysis (Perseus^®^, Martinsried, Germany). As a third step, proteins of similarly early upregulated clusters underwent further network analysis to evaluate possible corresponding proteins or functions. This approach, related to pooled proteomic data, is described in detail below.

### 2.6. Sample Preparation

The collected and stored serum samples were sent to OLINK (Analysis Service, Uppsala, Sweden) on dry ice for further proteomic analysis to allow for the high-quality and blinded proteomic analysis by a certified laboratory. The preparation was conducted according to their quality-checked protocol (ISO/IEC 17025:2005). Four internal controls were added to each sample to monitor the quality of the assay performance, as well as the quality of individual samples. The quality control (QC) is performed in two steps: Evaluation of each sample plate, based on the standard deviation of the internal controls, and the median value of the controls. Ninety percent of the samples passed for the OLINK inflammation panel, 95% for the neurology panel, and 100% for the organ damage panel.

### 2.7. OLINK Panels

Three OLINK panels were used for the analysis: Inflammation panel, organ damage panel, and neurology panel. For each protein, a unique pair of oligonucleotide-labelled antibody probes binds to the targeted protein, and if the two probes are close, a new PCR target sequence is formed by a proximity-dependent DNA polymerization event. The resulting sequence is subsequently detected and quantified using the standard real-time PCR. Then, the data are normalized and transformed using internal extension controls and inter-plate controls, to adjust for intra- and inter-run variation. The final assay read-out is given in normalized protein expression (NPX), which is an arbitrary unit on a log2 scale where a high value corresponds to the higher protein expression. Each proximity extension assay (PEA) measurement has a lower detection limit (LOD) calculated based on negative controls that are included in each run, and measurements below LOD were removed from further analysis. All of the assay characteristics, including detection limits and measurements of assay performance and validations, are available from the manufacturer’s webpage (http://www.olink.com, accessed on 14 December 2021). The analyses were based on 1 μL of serum for each panel of 92 assays [11]:

- The inflammation panel covers a wide range of inflammation-related protein biomarkers, which enables the analysis of 92 biomarkers through a multiplex immunoassay. The panel is assembled to detect an assortment of traditional, as well as exploratory, biomarkers within the inflammation research field.
- The organ damage panel investigates 92 biomarkers from 1 µL of the biological sample. It provides the optimal dynamic range and focuses on proteins that are relevant for processes involved in the biological response to organ damage. The proteins analysed in this panel are important in processes of response to stress, regulation of cell proliferation, cell cycle, and cell death/apoptosis.
- The neurology panel consists of a proximity extension assay (PEA) technology, which tests 92 neurology-related protein biomarkers across 96 samples simultaneously without compromising on data quality.

### 2.8. Bioinformatic Analysis of Proteins

After the protein expression analysis by OLINK, the identified and altered proteins were used for further bioinformatic investigations to classify underlying networks, signalling cascades, and affected pathways. Biological functions of regulated proteins were identified using the functional network analysis.

- Heatmapper (http://www.heatmapper.ca/, accessed on 14 December 2021) is an online server, which allows for the visualization of the results of gene expression profiling and cluster analysis in the form of heat maps through a graphical interface [12]. It allows for the accurate inspection of combinations of dataset characteristics to identify correlations and clustering results, as well as sample-related characteristics (e.g., survival time and gene expression levels). This approach allows for the visualization, as well as the accurate and rapid interpretation of the data obtained by large scale gene expression profiling [13]. By organizing complex data as matrix, the visualization of these data is improved. Heat mapping reorders rows and columns of the dataset to place the data with similar profiles, which are close to each other. In a second step, ranges of similar values are assigned to specific colour codes [14]. A heat map performs two actions on a matrix: First, it reorders the rows and columns to ensure that rows (and columns) with similar profiles are closer to one another, causing these profiles to be more visible. Second, each entry in the data matrix is displayed as a colour, making it possible to view the patterns graphically [14].
- GeneMANIA (http://www.genemania.org/, accessed on 14 December 2021) is a tool that helps in predicting the interactions and functions of a list of genes in a network form or when feasible, in pathways [15,16]. GeneMANIA provides the possibility of customizing the network, allowing for the choice of data sources or highlighting specific functions, with a more comfortable graphic experience [16]. GeneMANIA knowledge is based on data from large databases, which comprehend Gene Expression Omnibus, BioGRID, EMBL-EBI, Pfam, Ensembl, Mouse Genome Informatics, the National Center for Biotechnology Information, InParanoid, and Pathway Commons [15,16]. A network of interactions is created and the strength of the interaction is weighed. In the case of no interaction, an association weight of zero is assigned, while in the case of interaction, a positive value reflecting the strength of the interaction and the reliability of the finding, is assigned [17]. For example, the association of a pair of genes in a gene expression dataset is the Pearson correlation coefficient of their expression levels across multiple conditions in an experiment. The more the genes are co-expressed, the higher the weight they are linked by, ranging up to 1.0, indicating a perfectly correlated expression [15].
- WebGestalt is a tool to interpret the lists of genes from large scale x-OMICS (proteomics, genomics) studies [18]. The proteins of interest were uploaded to the tool where user IDs are unambiguously mapped to unique Entrez gene IDs, and all of them are mapped from a selected platform genome. Through the GoSlim classification plot, it is possible to examine the distribution of the genes of interest across the major branches of the gene ontology (GO) biological process, cellular component, and molecular function ontologies [19]. Each biological process, cellular component, and molecular function category is represented by a red, blue, and green bar, respectively.

### 2.9. Statistics

A *p*-value of < 0.05 was considered as statistically significant. For the analysis of demographic parameters, the U-test was utilised. For the protein expression analysis (OLINK data), the *t*-test was primarily utilised and supplemented with a Bonferroni correction to avoid the type I error due to multiple testing (*n* = 276 tests). In addition to statistical significance, the fold changes (FC) in protein regulation were analysed to address clinical relevance. Proteins with a fold change ≥2.0 and ≤−0.5 were considered clinically relevant and utilised for a second analysis approach.

The patients’ sample size was calculated using the *t*-test. From a preliminary set of patients and protein changes, as well as the assumption of an alpha error of 5% and a beta-error of 80%, adult patients (*n* = 20) were considered sufficient for the analysis.

### 2.10. Ethical Registration

This prospective observational cohort study was approved by the Ethics Committee of the University of Cologne, Faculty of Medicine, Cologne, Germany (No. 14-053) and was registered with ClinicalTrials.gov (Identifier: NCT02247947).

## 3. Results

A total number of patients (*n* = 20) were included in this study (Figure 1). The mean patient age was 69.9 ± 9.5 years (survivors: 60.9 ± 3.8 years; deceased: 69.2 ± 12.2 years; each, *n* = 10; *p* = 0.697; Table 1). All of the studied patients had a cardiac cause, which primarily led to cardiac arrest.

### 3.1. Proteomic Analysis

A total of 276 proteins were analysed with the three OLINK arrays and revealed 11 statistically significant protein alterations (neurology panel: *n* = 5 (proteins, Siglec-9, LAYN, SKR3, JAM-B, N2DL-2); inflammation panel: *n* = 1 (TNF-B); organ damage panel: *n* = 5 (BAMBI, NUCB2, STX8, PTK7, PVLAB)). After the application of Bonferroni correction, no proteins were found to be regulated as statistically significant. Concerning the protein fold change for clinical significance, a total of four proteins (IL-1 alpha, *n*-CDase, IL5, CRH) were found to be regulated with a fold change ≥2.0 or ≤−0.5.

### 3.2. Bioinformatic Analysis

Bioinformatic analysis was conducted on both groups of proteins with a significant *t*-test (prior to the Bonferroni correction) and to the group of proteins with a significant fold change.

**Figure 2:**
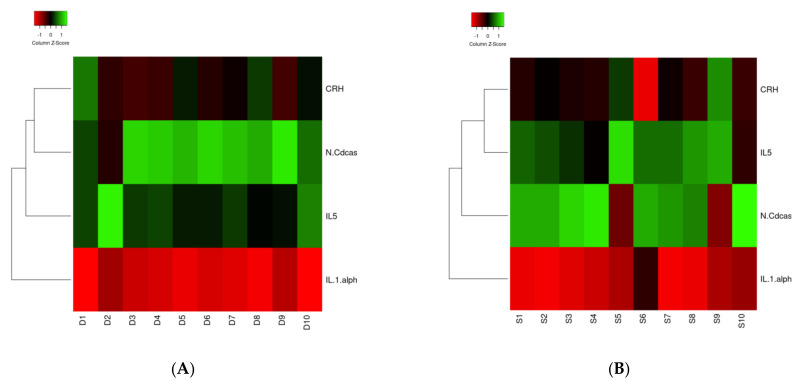

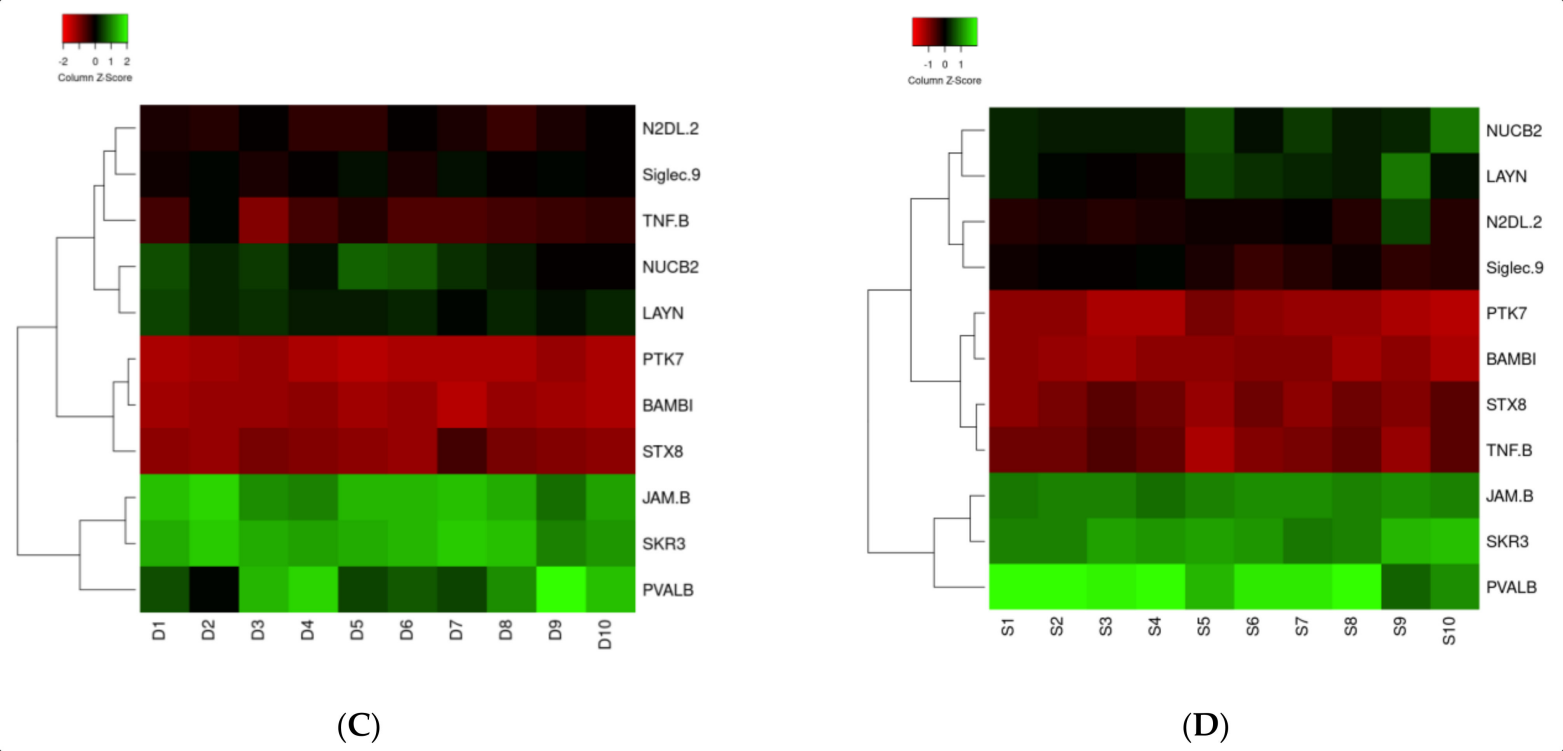
(**A**–**D**) Heat map of IL1A, ASAH2, IL5, and CRH ((**A**): Surviving patients; (**B**): Non-surviving patients) and TNF-B, Siglec-9, LAYN, SKR3, JAM-B, N2DL-2, BAMBI, NUCB2, STX8, PTK7, and PVALB ((**C**): Surviving patients; (**D**): Non-surviving patients) proteins in correlation with the survival and dead groups. The 11 proteins in the middle were found in the present study. Circulated proteins were identified as linked to the proteins of the present study, which is shown by the red lines.

Heat map analysis for the four clinically relevant proteins in survivors and non-survivors showed no difference in clustering (Figure 2A,B). IL1A was downregulated, and CRH, IL5, and *n*-CDAS were upregulated in both groups. Concerning the analysis of the 11 statically significant proteins, clustering for regulation was different for the proteins in surviving and non-surviving patients (Figure 2C,D). Solely clustering of TNF-B was different between both groups and was allocated to another cluster.

**Figure 3:**
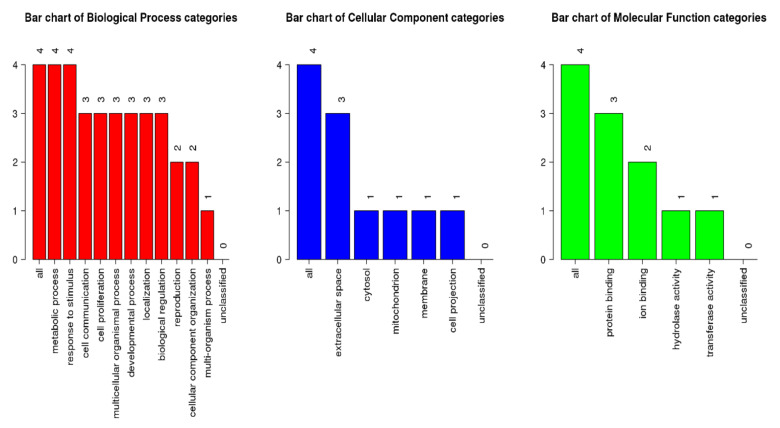
GOslim summary for the biological process, cellular component, and molecular function category for IL1A, ASAH2, IL5, and CRH proteins. Each of the functions is represented by a red, blue, and green bar, respectively.

From WebGestalt, all four proteins with >2/<−0.5-fold changes were shown to be involved in metabolic processes, response to stimuli, and cell communication (biological process category) (Figure 3). Three proteins were involved in extracellular space (cellular component category) and protein binding (molecular function category). The GeneMania software was utilized to examine the network and correlating proteins for each group.

**Figure 4:**
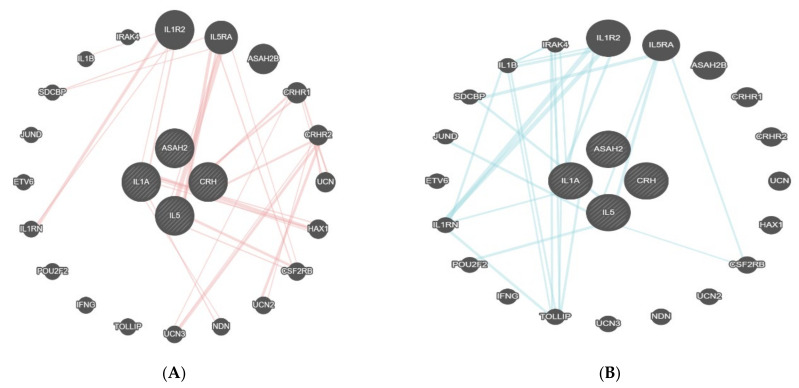
(**A**,**B**) Physical interaction table of IL1A, ASAH2, IL5, and CRH proteins. This figure shows the connection between the proteins found in the present analysis (within the circle) and the linked proteins as identified by GeneMania. The four proteins in the centre were found in the present study. All of the other encircled proteins in the periphery linked with red lines to the central proteins are the ones with a relevant physical interaction with the latter.

Interleukin-1-alpha (IL1A), *n*-acylsphingosine amidohydrolase-2 (ASAH2), corticotropin-releasing hormone (CRH), and interleukin-5 (IL5) showed physical interactions with interleukin-1 receptor type 2 (IL1R2), interleukin-5 receptor subunit alpha (IL5RA), corticotropin-releasing hormone receptor-1 (CRHR1), corticotropin-releasing hormone receptor-2 (CRHR2), and other proteins (Figure 4A). Additionally, the pathway interaction of these proteins was studied. IL1A and IL5, in their pathways, present several important proteins, such as IL1R2, IL5RA, interleukin-1 receptor-associated kinase 4 (IRAK4), and cytokine receptor common subunit beta (CSF2RB) (Figure 4B).

**Figure 5:**
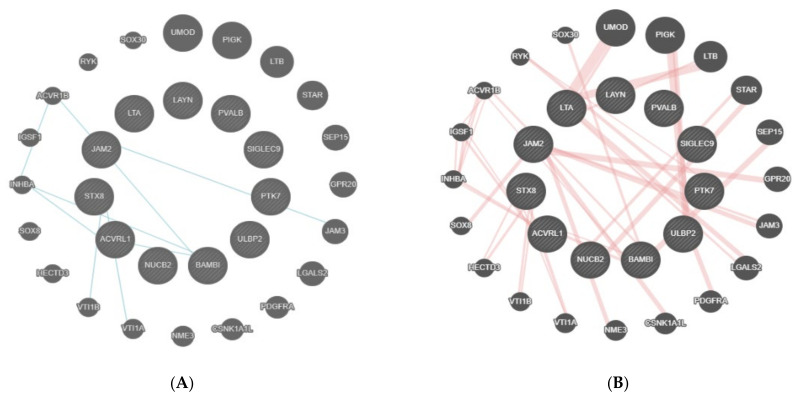
(**A**) shows the physical interaction table for TNF-B, Siglec-9, LAYN, SKR3, JAM-B, N2DL-2, BAMBI, NUCB2, STX8, PTK7, and PVALB. This figure shows the connection between the proteins found in the present analysis (within the circle) and the linked proteins as identified by GeneMania. The 11 central proteins were identified in the present study. All of the other encircled proteins in the periphery linked with blue lines to the central proteins are the ones with a relevant physical interaction with the latter. Interaction of proteins is visualized by blue lines. (**B**) shows pathway interactions for TNF-B, Siglec-9, LAYN, SKR3, JAM-B, N2DL-2, BAMBI, NUCB2, STX8, PTK7, and PVALB. The 11 proteins in the middle were found in the present study. The circulated proteins were identified as linked to the proteins of the present study, which is shown by the red lines.

After the bioinformatic analysis, the 11 proteins that were found as statistically significant (*t*-test) had pathway interactions only with a few proteins, such as activin receptor type-1B (ACVR1B), junctional adhesion molecule 3 (JAM3), vesicle transport through interaction with T-SNAREs 1A and IB (VTI1A VTI1B), and inhibin subunit beta A (INHBA) (Figure 5A). The physical interactions of these genes included links with proteins, such as uromodulin (UMOD), phosphatidylinositol glycan anchor biosynthesis class K LTB (PIGK), SRY-box transcription factor 30 (SOX30), etc. (Figure 5B).

## 4. Discussion

The aim of the present study was to identify protein biomarkers to facilitate the prognostication for survival in OHCA patients after CPR, ROSC, and TTM. Of the 276 proteins analysed from the three OLINK panels, four showed a clinically relevant regulation and 11 proteins showed statistical significance. However, after Bonferroni correction, the statistical significance was no longer demonstrated. Bioinformatic analysis revealed the pathways involved and the related proteins which were significantly altered.

### 4.1. Patient Population

For the present study, the patient group was dichotomized into surviving and non-surviving patients for the investigation of specific protein regulation patterns in each respective group. Since survival is most often used as a hard outcome parameter after CPR [20,21], it was chosen to separate the patient groups.

Concerning the demographic parameters of the patients, the ages of the patients were comparable between the survivors and non-survivors (70.8 vs. 69.2 years) and the mean age (69.9 years) is comparable to the age of patients that are presented in other papers regarding cardiac arrest and CPR [22,23]. In the present study, at least the age of the patients indicates that the patient group may be comparable to the other patients. However, the male:female proportion (19:1) is significantly different and gender aspects seem of low relevance for this specific aspect of protein expression. Nevertheless, the role of gender aspects seems to be controversially discussed [24].

### 4.2. Protein Identification

In the present study, four vs. 11 proteins were found to have a significantly different serum expression to discriminate survivors and non-survivors. Although significance was not achieved after Bonferroni correction, the proteins are of interest for a clinically relevant approach (fold >2 and <−0.5). The aim of this study was not to find single biomarkers for a definite answer, but a full set or array of proteins which could facilitate prognostication.

Of all the proteins found to be significantly regulated in the present study, TNFB seems to be most promising. TNFB was significantly downregulated in the non-survivors’ group, which could be an early indicator of low survival. However, from the cluster analysis of all the other proteins analysed, up and downregulation was comparable on both groups.

In addition to the findings of the present study, a recent trial from Cakmak et al. [25] found that serum copeptin levels predict ROSC and the short-term prognosis of patients with OHCA. Therefore, the authors concluded that serum copeptin levels may serve as a guide in diagnostic decision making to predict ROSC in patients undergoing CPR and in determining the short-term prognosis of patients with ROSC. In this study, blood was drawn at patient admission. However, the aim of the present study was to identify protein alteration with a potential to predict the overall outcome after TTM, for use in a later protein expression profile.

### 4.3. Biological Processes and Cascades

Concerning the biological function and the associated cascades, proteins were mainly involved in the metabolic process and biological regulation with a protein binding function. In addition, the proteins originated from the membrane and extracellular space. Although this indication does not directly reveal the relevant proteins, it may give some suggestion for identifying the important proteins in this context. Since the present study was not designed to find these new or unknown proteins, it can provide a suggestion for which other proteins may be interesting for analysis in future studies. Moreover, this evidence provides insight into the function of the identified proteins in the metabolic and cellular processes, which might be relevantly affected, and consequently require attention for treatment.

### 4.4. Limitations

In the present study, three different panels, with each containing 96 different proteins, were used for the analysis. For a careful interpretation of the results, several limitations are noteworthy. First, the approach utilised specific proteins and not a broad analysis of potentially unknown proteins. Therefore, it was not possible to identify new biomarkers for prognostication. Second, this can be considered only as a first pilot study for future analysis, and only patients (*n* = 20) were examined. Potentially, if several hundred or thousands of patients are analysed, additional markers could be found. This study could be a solid base for future clinical studies, even if it has specific limitations.

## 5. Conclusions

The present study aimed to identify proteins associated with survival or death at day 30 after out-of-hospital cardiac arrest and ROSC. Although several proteins were identified to reveal statistical or clinical relevance, bioinformatic analyses unveiled no promising candidates. Therefore, early analysis at 24 h after ROSC was not sufficiently possible during TTM to prognosticate survival or non-survival after cardiac-induced OHCA. As a result, further studies are necessary to better evaluate protein expression after ROSC and to identify promising protein candidates for prognostication, e.g., 6 h post-ROSC. This study could be considered as a launching platform for future multi-centric studies.

## Figures and Tables

**Table 1:** Demographics table reporting on the different analysed variables. All of the parameters are given as means +/−SD. For the analysis of demographic parameters, the U-test was utilised.

	Total	Survived	Dead	*p*-Value
Number of patients	*n* = 20	*n* = 10	*n* =10	
Age	69.9 (9.5)	70.8 (3.8)	69.2 (12.3)	0.697
Gender				<0.001
Female	1 (5%)	0 (0%)	1 (10%)	
Male	19 (95%)	10 (100%)	9 (90%)	
Cause of cardiac arrest				
Myocardial infarction–no. (%)	14 (70)	8 (80)	6 (60)	0.620
Primary arrhythmia–no. (%)	6 (30)	2 (20)	4 (40)	0.620
Cardiac arrest characteristics				
No-flow time (min)	4.2 ± 2.9	3.0 ± 1.7	5.4 ± 3.6	0.030
Witnessed arrest by bystander–no. (%)	14 (70)	9 (90)	5 (50)	0.140
BLS provided by bystander–no. (%)	15 (75)	8 (80)	7 (70)	0.990
Dose of epinephrine during CPR (mg)	5.5 ± 4.2	4.8 ± 4.1	6.0 ± 4.2	0.060
Number of shocks	5.0 ± 4.2	4.7 ± 4.1	5.3 ± 4.3	0.730
Time to ROSC (min)	17 ± 11	15 ± 12	23 ± 15	0.080
ICU treatment				
Period of ICU hospitalization (days)	12 ± 12	14 ± 9	7 ± 6	0.04
Ventilation time (days)	11 ± 10	10 ± 12	7 ± 5	0.07

## Abbreviations

ACVR1B	Activin receptor type-1B
ASAH2	*N*-acylsphingosine amidohydrolase-2
BAMBI	Activin membrane-bound inhibitor homolog
CCT	Cranial computer tomography
CPR	Cardiopulmonary resuscitation
CRH	Corticotropin-releasing hormone
CRHR1	Corticotropin-releasing hormone receptor-1
CRHR2	Corticotropin-releasing hormone receptor-2
CSF2RB	Cytokine receptor common subunit beta
DNA	Desoxyribonucleid acid
EEG	Electroencephalography
ERC	European resuscitation council
FC	Fold change
GO	Gene ontology
HIBI	Hypoxic−ischemic brain injury
ICU	Intensive care unit
IL-1A	Interleukin-1-alpha
IL1R2	Interleukin-1 receptor type 2
IL5	Interleukin-5
IL5RA	Interleukin-5 receptor subunit alpha
INHBA	Inhibin subunit beta A
IRAK4	Interleukin-1 receptor-associated kinase 4
JAM-B	Junctional adhesion molecule B
JAM3	Junctional adhesion molecule 3
LAYN	Layilin
LOD	Lower detection limit
NPX	Normalized protein expression
NSE	Enolase-2
NUCB2	Nucleobindin-2
N2DL-2	NKG2D ligand-2
*n*-CDase	Neutral ceramidase
OHCA	Out-of-hospital cardiac arrest
PCR	Polymerase chain reaction
PEA	Proximity extension assay
PIGK	Phosphatidylinositol glycan anchor biosynthesis class K LTB
PT	Prothrombin time
PTK7	Tyrosin protein kinase 7
PTT	Partial thromboplastin time
QC	Quality control
ROSC	Return of spontaneous circulation
SEP	Somatosensory-evocable potentials
Siglec-9	Sialic acid-binding immunoglobulin-like lectin 9
SOP	Standard operating procedure
SOX30	SRY-box transcription factor 30
STX8	Syntaxin 8
TNF-B	Tumour necrosis factor
TTM	Targeted temperature management
UMOD	Uromodulin
VTI1A	Vesicle transport through interaction with T-SNAREs 1A
VTI1B	Vesicle transport through interaction with T-SNAREs 1B
